# The Ethics of Ante-Mortem Stimulation

**Published:** 1914-12

**Authors:** 


### The Ethics of Ante-Mortem Stimulation.

Our attention has been called to this subject by a most interesting article on the Treatment of Somatic Death, by W. Wayne Babcock of Philadelphia, in the "Monthly Cyclopedia." Cardiac massage, external and by fingers introduced through intercostal punctures, intravenous injections of saline solution and drugs, artificial respiration by external measures and intubation, etc., were employed. Of the eight reported cases, two resulted in permanent recovery; the rest included two failures, one maintenance of respiration in shock for six hours, three postponements in inevitable death for periods of a few days.

In 1901, we were called to a feeble man of about 65, who had hepatic sclerosis and arteriosclerosis and who, a few years previously, had apparently recovered from incipient phthisis. He was apparently dead from syncope due to exercise in the hot sun. In spite of the protest of his wife who regarded the procedure as an indignity to the remains, hot saline was injected into the bowel—really a more prompt method than hypodermoclysis—and the patient recovered and is still alive though his condition for the last six years has been so wretched as to lead to some regret.

In another instance, judged to be absolutely hopeless on account of hepatic sclerosis and advanced renal degeneration, attempts at stimulation had been abandoned, as a matter of mercy. A consultant insisted on stimulation. At this time, respiration and heart beat had just ceased but both were almostly instantly restored by a hypodermic of 3 milligrams of strychnine—an interesting observation on account of the scepticism recently manifested regarding this drug. But the revival endured only a few minutes.

Perhaps on account of a central location and an arrangement of office hours intended to accommodate a non-emergent practice, the writer has had an unexpected number of calls in cases of actual or apparent death, as well as the usual problem of stimulation in desperate cases of diseases, more or less hopeless. It would be out of place in an editorial to discuss the scientific diagnostic, prognostic and therapeutic problems of threatened or doubtful death, but certain ethical problems of a more general nature remain. As the logic by which these problems is decided involves the personal equation to a large degree, and as the nature of the argument in each case is obvious, time may be saved by the statement of conclusions, but with no intention of being dogmatic nor of contending that these conclusions represent the ultimate ethical truth. Indeed, criticism is asked, as the problems are of the utmost importance and as they are placed before both experienced and inexperienced members of the profession, under conditions that allow no time for careful consideration.

1. In a case of (apparent) sudden death of a person whom the physician has not known or of whose physical condition the physician has no knowledge, prompt stimulation should be administered, without delaying to determine whether death is real or apparent.

2. In either acute or chronic disease, including shock and traumatisms, in the absence of factors absolutely precluding hope of recovery or of postponement of death for a considerable period, stimulation should be maintained up to the time at which somatic death is established and indeed, for a considerable time after cessation of pulse and respiration.

3. In malignant disease, incurable visceral lesions as of the heart, lungs, kidney, liver and pancreas and brain, etc., in which the case has been carefully studied and the maintenance of life is obviously impossible, stimulation should not be carried out simply to prolong life for a few minutes or hours, unless:

4. There is some strong religious, business or sentimental reason for temporarily postponing death, such as the administration of the sacrament, the making of a will, the delayed arrival of a member of the family. A curious ethical problem was involved in the following case of which we have no personal knowledge: A man and his wife lay at the point of death, there being no possibility of revising wills. The survival of either for even a moment beyond the other would determine the disposition of a considerable property. Each set of relatives employed a physician to stimulate the patient for purely mercenary motives. What would be the duty of a physician summoned to prolong life under such conditions?

5. Every physician,—without regard to limitation of practice or retirement—should be prepared to render emergent aid. The nature of the stimulation in case of impending or probable death, should, of course, be based on thorough scientific study, but preference should be given to methods capable of prompt application on the spot, rather than to methods theoretically superior but impracticable.

6. The mere desirability of death, either from the standpoint of the patient or of relatives or of the community, should have no influence upon the physician.

7. Aside from the scientific interest, there is a strong ethical need of determination of reliable and immediately applicable tests of death.

